# Etiology of Childhood Infectious Diarrhea in a Developed Region of China: Compared to Childhood Diarrhea in a Developing Region and Adult Diarrhea in a Developed Region

**DOI:** 10.1371/journal.pone.0142136

**Published:** 2015-11-03

**Authors:** Xin Wang, Jing Wang, Hao Sun, Shengli Xia, Ran Duan, Junrong Liang, Yuchun Xiao, Haiyan Qiu, Guangliang Shan, Huaiqi Jing

**Affiliations:** 1 Institute of Basic Medicine Science, Chinese Academy of Medicine Sciences, School of Basic Medicine, Peking Union Medicine College, Beijing, China; 2 National Institute for Communicable Disease Control and Prevention, Chinese Centre for Disease Control and Prevention, State Key Laboratory for Infectious Disease Prevention and Control, Collaborative Innovation Center for Diagnosis and Treatment of Infectious Diseases, Beijing, China; 3 Department of Infectious Disease, Dongcheng Centre for Disease Control and Prevention, Beijing, China; 4 Department of Infectious Disease, Henan Provincial Centre for Disease Control and Prevention, Zhengzhou, China; University of Texas Medical Branch, UNITED STATES

## Abstract

In China, great differences in economy, social characteristics and hygiene exist between developing and developed regions. A comparative study of infectious diarrhea between two regions was needed. Three groups of diarrheal patients were collected: children ≤5 year-olds from Beijing (developed region) and Henan Province (developing region), and adults over 18 year-olds from Beijing. A questionnaire was used to survey and feces samples were examined for 16 enteropathogens. We enrolled 1422 children and 1047 adults from developed region and 755 children from developing region. Virus positive rates were 32.98% for children and 23.67% for adults in developed region. The most prevalent pathogen for children was rotavirus whereas for adults was norovirus. Bacterial isolation rates were 13.92% for children from developed region, while 29.14% for children from the developing regions. For the greatest difference, *Shigella* accounted for 50.79% and was the dominant pathogen in the developing region, whereas in the developed region it was only 1.45%. There was no significant relationship between the local levels of development with diarrheogenic *Escherichia coli* (DEC) categories. But it was seen the notable differences between the population with different age: enteropathogenic *E*.*coli* (EPEC) and enteroaggregative *E*.*coli* (EAggEC) were the primary classes of DEC in children from both regions, whereas it was enterotoxigenic *E*.*coli* (ETEC) in adults. The symptoms of *Shigella* and *Salmonella* infection, such as bloody stools, white blood cells (WBC) and red blood cells (RBC) positivity and fever were similar in children, which may lead to the misidentification. *Yersinia enterocolitica* and shiga toxin-producing *E*.*coli* (STEC) infections were firstly reported in Beijing. There was a large difference in etiology of bacterial diarrhea between children in developing and developed regions of China.

## Introduction

Infectious diarrhea is a major concern for public health worldwide [1] and is caused by water and food contaminated with pathogenic bacteria, viruses, or parasites [2]. Diarrheal disease is the primary cause of morbidity and mortality among children in developing countries [1, 3]. The etiology of infectious diarrhea has obvious divergence among regions with different economic and hygiene development [4]. The common bacteria causing diarrhea in developed countries are *Salmonella*, *Campylobacter*, shiga toxin-producing *E*.*coli* (STEC), enteropathogenic *E*.*coli* (EPEC), and enteroaggregative *E*.*coli* (EAggEC) in the United States [5], Greece [6], Denmark [7], France [8], and the United Kingdom [3]. *Shigella* and enterotoxigenic *E*.*coli* (ETEC) are frequently found among developing countries, as sub-Saharan Africans, south Asians [1, 9, 10], south Americans [11], Pacific Islanders [12, 13]. The etiology of viral diarrhea is similar between developed and developing countries [4, 8, 12]. Etiology surveys of infectious diarrhea in China are limited [14–16], especially for bacteria etiology and also for detecting virus and bacteria simultaneously. Considering etiology differences of infectious diarrhea between children in developed and developing regions of China was unclear, we studied children with diarrhea who were no more than 5 years old from a community children hospital in Beijing for three years consecutively; and recruited diarrheal adults over 18 years old from a general hospital in Beijing and diarrheal children in a village clinic in Henan Province as a control.

## Materials and Methods

### Population Design and Investigation

All the cases enrolled and performed as the same protocol. The diarrhea was defined as three or more loose stools within the previous 24hrs according to the Global Enteric Multicenter Study (GEMS) [1]. Beijing City and Henan province, the typical developed and developing regions of China, were selected to do the study. The diarrhea outpatients of no more than 5 years old at a community pediatric hospital were recruited from 1 October 2011 to 30 September 2014 in city center of Beijing, representative of a developed region. To make a pathogen spectrum comparison, adult patients over 18 years old were collected from the enteric clinic of a general hospital in Beijing during 2013 to 2014 in Beijing; and childhood patients of the same age range were collected from a village clinic from January 2010 to December 2014 in the poor countryside in Sui County of Henan Province, representative of a developing region. A questionnaire to each case was used to survey demographics (name, gender, birthday, address, contact information) and clinical features (the date of onset, the date of visiting doctor, diarrhea frequency, body temperature, vomiting, fecal property and results of stool routine inspection). The routine examination of the stools was performed to detect the presence of white blood cells (WBC) and red blood cells (RBC) in the stool samples. For the limitations of village clinic, stool routine inspection didn’t be examined in Sui county of Henan.

### Detection of Pathogens

#### Samples

Fresh stool were collected when the cases visited the doctor and enrolled to this study, these were analysed to detect five viruses (rotavirus, norovirus, sapovirus, astrovirus, and adenovirus) and 11 bacteria (*Salmonella*, *Shigella*, diarrheogenic *Escherichia coli* [DEC], *Vibrio cholera*, *Vibrio parahaemolyticus*, *Yersinia enterocolitica*, *Yersinia pseudotuberculosis*, *Campylobacter jejuni*, *Campylobacter coli*, *Aeromonas hydrophila*, and *Plesimonas shigelloides*) in the pathogen laboratories of centre for disease control and prevention. The same detection procedure was performed at each site.

Samples from Beijing childhood cases were examined for all bacteria and viruses. Sample of Beijing adults were tested for 11 bacteria in the year of 2013 and for all bacteria and viruses both in the year of 2014. Sample from Sui County of Henan were tested for 11 bacteria. Previous studies have shown that there are no significant differences between viral diarrhea in children from developed and developing regions [4, 8, 12], which was supported by an unpublished Chinese national survey either. Virus detection was conducted in the developed region but not in the Henan countryside due to lack of suitable laboratory facilities.

#### Viruses Detection

Samples for the virus detection were placed in the commercial virus sampling tube and storing and transferred to the pathogen laboratory at the temperature lower than -20°C. Viral DNA or RNA was extracted from sample (QIAGEN, USA) and the first strand cDNAs were synthesized from the extract viral RNAs. The multiplex PCR was performed to detect rotavirus, norovirus, sapovirus, astrovirus, and adenovirus. Further, PCR was performed for subtyping rotavirus (groups A, B, and C) and norovirus (GI and GII). The detection of virus within the samples was performed as previously described [17, 18] (The PCR primers for each virus were showed in S1 Table).

#### Bacteria isolation

Enough samples for the bacterial tests were collected and placed in Carry-Blair medium and transferred to the laboratory at 4°C within 24 hours to allow the isolation and identification of the bacteria described above. To isolate *Salmonella* [19], the samples were placed into selenite brilliant green (SBG) sulfa enrichment broth (BD, USA) and incubated at 37°C for 16h. The inoculate was then placed onto the *Salmonella-Shigella* (SS) agar or CHROMagar *Salmonella* Medium (CHROMagar, France) at 37°C overnight, selected the suspicious colonies to perform ortho-nitrophenyl-beta-D- galactopyranoside (ONPG) test, and finally confirmed strains by Api20E (bioMérieux, France). To isolate *Shigella*, sample were streaked onto the *Salmonella-Shigella* (SS) agar, MacConkey (MAC) agar or xylose lysine desoxycholate (XLD) agar, incubated at 37°C for 16-24h and chose the suspicious colonies to test biochemical reactions by Kligler iron agar (KIA) and motility indole urea semisolid medium (MIU). Identified *Shigella* strain and the serotype by antisera of *Shigella*. To isolate diarrheogenic *Escherichia coli* (DEC), the samples were inoculated onto MAC agar, incubated at 37°C for 16-24h and selected the suspicious colonies to perform biochemical reactions by KIA, MIU and Indole/Methyl red/Voges—Proskauer/Citrate (IMViC) test to identify suspicious presumptive *E*.*coli* strains. The detection of the virulence genes of suspicious presumptive *E*.*coli* strains was performed by multiplex PCR, as described previously in [20] (The PCR primers were showed in S2 Table) to identify EPEC, EAggEC, ETEC, enteroinvasive *E*. *coli* (EIEC) and STEC. To isolate *V*.*cholera*, *V*.*parahaemolyticus*, *A*.*hydrophila*, and *P*.*shigelloides*, the samples were cultured by alkaline peptone water (AWP) at 37°C for 6-8h and inoculated on thiosulfate citrate bile salts sucrose (TCBS) agar (BD, USA), MAC agar and blood plate. The presumptive colonies were examined for oxidase activity and positive isolates were identified by the Api20E/NE test Api20E/NE (bioMérieux, France). To isolate *Y*.*enterocolitica* and *Y*.*pseudotuberculosis* [21], enrichment was performed by using peptone sorbitol bile broth(Fluka, USA) at 4°C for 10–20 days, and then the strains were inoculated onto *Yersinia* selective agar (cefsulodin-Irgasan-novobiocin [CIN] agar) (BD, USA) and incubated at 25°C for 24h. Colonies were selected by KIA and MIU and identified by Api20E (bioMérieux, France). To isolate *C*.*jejuni* and *C*.*coli*, the samples were inoculated Skirrow selective medium (BD, USA)which added blood and incubated at 42°C in microaerophilic environment for 2-3days and the suspicious strains were identified following the oxidase, catalytic and hippurate hydrolysis tests.

### Data Analysis

Statistical analysis was performed using Stata software, version 12.0. Isolation rates from adults and children were compared using the Chi-square method, and adjusted for confounders and season. In the comparison of the urban and rural children, age group and season were adjusted.

### Ethics

This study was approved by the ethics review committee [Institutional Review Board (IRB)] of National Institute for Communicable Disease Control and Prevention, Chinese Center for Disease Control and Prevention. Signed informed consent was obtained from all study participants. For all the patients under 18 years-old, a written consent form was signed by a parent or legal guardian.

## Results

### Characteristics of Cases

#### Demography of Subjects

During the period from 1 October 2011 to 30 September 2014, a total of 1422 outpatients aged 5 years or under were enrolled from the developed region. In the adult group from the developed region, 1047 adult cases were collected from March 2013 to October 2014, excluding November to February because the enteric clinic is closed during this period according to Chinese regulation. In the group from the developing region, 755 children cases were enrolled from January 2010 to December 2014. Childhood diarrhea throughout the year demonstrated the peak season for episodes to be in the summer and autumn in both of the developed and developing regions. In the adult population from developed region the episodes of diarrhea were found in the spring and autumn but the highest levels were detected in the summer (Table 1).

**Table 1:** Demographic and clinical symptoms of cases.

Characteristics		No. of enrolled cases (proportion %)			
		Children in developed region	Children in developing region	Adults in developed region	
Gender	Male	841(59.14)	504(66.75)		503(48.04)
	Female	581(40.86)	251(33.25)		544(51.96)
Age(years)	0-	589(41.42)	220(29.14)	18-	117(11.17)
	1-	494(34.74)	312(41.32)	25-	409(39.06)
	2-	151(10.62)	136(18.01)	35-	140(13.37)
	3-	95(6.68)	48(6.36)	45-	131(12.51)
	4-	53(3.73)	27(3.58)	55-	115(10.98)
	5-	40(2.81)	12(1.59)	65-	135(12.89)
Season	Spring(Mar-May)	254(17.86)	87(11.52)		290(27.70)
	Summer(Jun-Aug)	571(40.15)	297(39.34)		491(46.89)
	Autumn(Sep-Nov)	420(29.54)	321(42.52)		266(25.41)
	Winter(Dec-Feb)	177(12.45)	50(6.62)		-
Clinical Symptoms	Diarrhea frequency (times/day Mean±SD)	4.35±1.72	6.15±2.15		5.69±2.23
	Fever(>37.2℃)	130(9.14)	195(25.83)		13(1.24)
	Temperature(℃)	38.46±0.62	38.33±0.68		38.55±0.54
	Vomit	156(10.97)	193(25.56)		279(26.65)
Fecal property	Watery	883(58.58)	156(20.66)		633(60.46)
	Mucus	29(2.04)	136(18.01)		26(2.48)
	Bloody	13(0.91)	356(47.15)		5(0.48)
	Loose	497(34.95)	107(14.17)		383(36.58)
Stool Routine^a^	WBC-	929(65.33)	-		492(46.99)
	WBC+	488(34.42)	-		555(53.01)
	RBC-	1326(93.25)	-		265(25.31)
	RBC+	91(6.40)	-		782(74.69)

^a^: 1417 childhood cases were examined the stool routine inspection in developed region.

#### Clinical Features

Symptoms of diarrhea frequency, excrement character, fever, vomiting, and test results for WBC and RBC are shown in Table 1. Children no more than 5 years old in the developed regions had lower average diarrhea frequency (per day) than those in the developing regions. Watery stools predominated in the developed regions (children 58.58%, adults 60.46%), whereas bloody stools were found in the developing region (47.15%). Clinical symptoms were the most severe in children from the developing region, followed by adults and children from the developed region.

### Pathogen Spectrums from Diarrheal Children between Developed and Developing Regions

**Fig 1:**
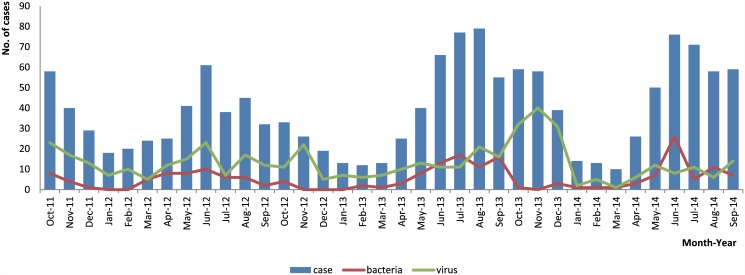
Monthly number of enrolled, virus positive and bacteria isolated cases of children in the developed region.

A total of 469 cases were viral positive and 198 cases isolated strains, which was belonged to 11 tested bacteria, from 1422 childhood cases of developed region. The time trend of cases enrolled, bacteria positive and virus positive were showed in Fig 1. And bacteria were isolated 220 cases from the developing region (Table 2). Onset season and age group were significantly associated with isolation rates in both regions, so they were adjusted in order to compare the isolation rates. Total bacteria, *Shigella* and *A*.*hydrophila* isolation rates showed significant differences between developed and developing regions (Table 2). Multivariable regression analysis including covariates gender, age group, onset season, and regions showed that the *Shigella* isolation rate in developing regions was 88.59 times higher compared to that in the developed region (OR = 88.59; 95% confidence interval [CI]:[27.97,-280.56]; *P* = 0.000) and *A*.*hydrophila* was 5.55 times higher(OR = 5.55, 95%CI:[2.38–12.96]; *P* = 0.000).

**Table 2:** Comparison of the positive rate of childhood cases in the developed region with adult cases in the developed region and childhood cases in the developing region.

Pathogen		Children in Developed region (n = 1422)		Adults in Developed region ^a^ (n1 = 1047; n2 = 507)		Children in Developing region (n = 755)		*P* value of comparison between the childhood and adult cases in the developed regions		*P* value of comparison between the childhood cases in the developed and developing regions		
		No. of positive	positive rate(%)	No. of positive	positive rate(%)	No. of isolates	positive rate(%)	*P*	*P* (Adjust by season)	*P*	*P* (Adjust by age)	*P* (Adjust by season)
Bacteria		198	13.92	186	17.77	220	29.14	0.009	0.000	0.000	0.000	0.000
	*Salmonella*	61	4.29	35	3.34	40	5.30	0.229	0.369	0.287	0.357	0.297
	*Shigella*	3	0.21	15	1.43	128	16.95	0.000	0.001	0.000	0.000	0.000
	DEC	128	9.00	111	10.60	54	7.15	0.184	0.824	0.138	0.044	0.111
	EAggEC	50	3.52	18	1.72	22	2.91	0.007	0.000	0.455	0.161	0.320
	EPEC	64	4.50	22	2.10	27	3.58	0.001	0.000	0.305	0.291	0.367
	ETEC	5	0.35	65	6.21	2	0.26	0.000	0.000	0.734	0.741	0.749
	EIEC	5	0.35	5	0.48	1	0.13	0.626	0.665	0.353	0.290	0.294
	STEC	5	0.35	1	0.10	2	0.26	0.201	0.145	0.734	0.606	0.700
	*Vibrio* ^b^	1	0.07	25	2.39	0	0.00	0.000	0.000	0.466	0.541	0.471
	*Yersinia* ^c^	7	0.49	0	0.00	2	0.26	0.023	0.014	0.431	0.230	0.539
	*Campylobacter* ^d^	0	0.00	0	0.00	1	0.13	-	-	0.170	0.102	0.166
	*Aeromonas hydrophila*	7	0.49	7	0.67	27	3.58	0.564	0.651	0.000	0.000	0.000
	*Plesimonas shigelloides*	0	0.00	3	0.29	0	0.00	0.043	0.062	-	-	-
Virus		469	32.98	120	23.67	-	-	0.000	0.010			
	Rotavirus^^e^^	179	12.59	21	4.14	-	-	0.000	0.001			
	Norovirus	139	9.77	57	11.24	-	-	0.314	0.248			
	Sapovirus	64	4.50	20	3.74	-	-	0.626	0.755			
	Astrovirus	48	3.38	25	4.93	-	-	0.106	0.039			
	Adenovirus	93	6.54	4	0.79	-	-	0.000	0.000			

^a^: n1: Total number of cases detecting bacteria; n2:Number of cases detecting virus and bacteria both.

^b^: *Vibrio cholera* and *Vibrio parahaemolyticus*.

^c^: *Yersinia enterocolitica* and *Yersinia pseudotuberculosis*.

^d^: *Campylobacter jejuni* and *Campylobacter coli*.

^e^: All of the positive cases were Rotavirus Group A.

**Fig 2:**
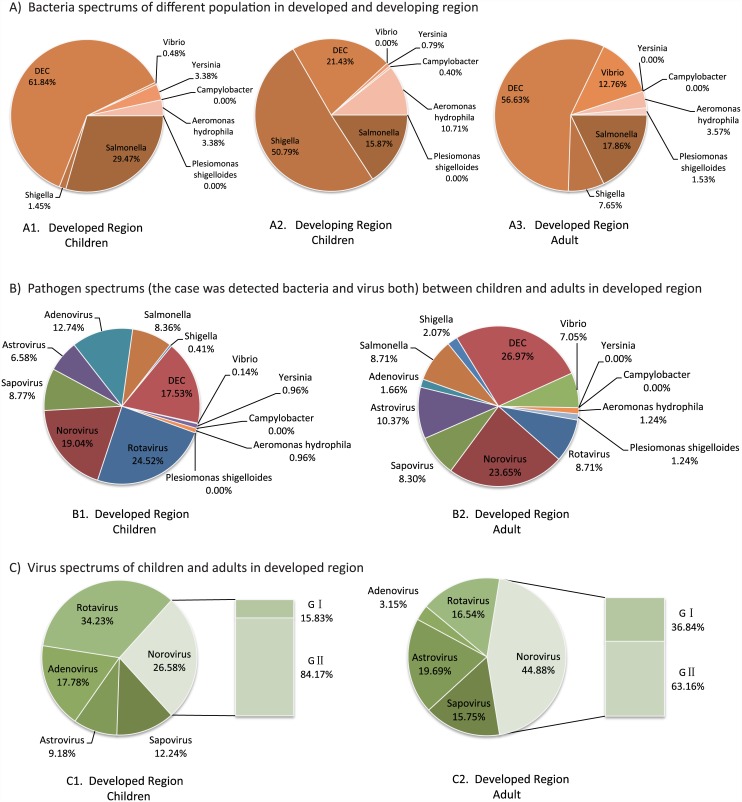
Pathogen Spectrum of cases in the developed and developing region (%: proportion). A1. Bacteria spectrum of childhood cases in the developed region; A2: Bacteria spectrum of childhood cases in the developing region; A3: Bacteria spectrum of adult cases in the developed region; B1. Pathogens (one case was detected bacteria and virus both) spectrum of childhood cases in the developed region; B2: Pathogens (one case was detected bacteria and virus both) spectrum of adult cases in the developed region. C1. Virus spectrum of childhood cases in the developed region; C2: Virus spectrum of adult cases in the developed region.

For the pathogen spectrum in sub-developed region children and developed region children, *Shigella* accounted for 50.79% and was the dominant pathogen in the developing region, whereas in the developed region it was only 1.45%. The DEC proportion in the developing region was one-third that of the developed region and *Salmonella* was half. *A*.*hydrophila* accounts for 10.71% in the developing region—three times that in the developed region (Fig 2A1 and 2A2).

**Fig 3:**
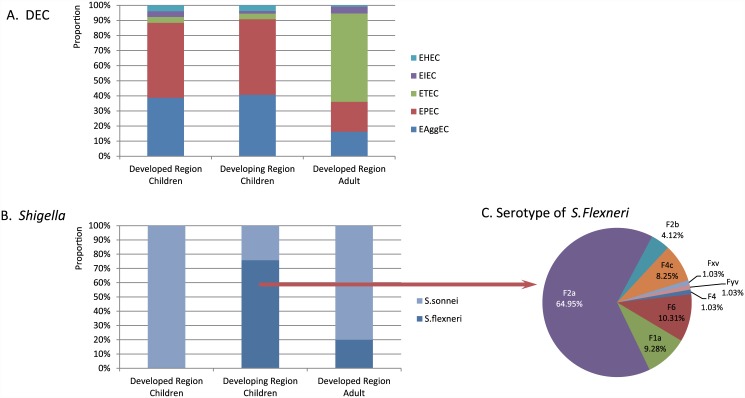
The proportion of five classes of DEC and serum groups of *Shigella*. A. The proportion of five classes of DEC; B: The proportion of serum groups of *Shigella*; C: The proportion of type of *S*.*Flexneri*.

*Shigella sonnei* comprised 24.22% of the 128 *Shigella* strains isolated in the developing region, with the remainder being *S*. *flexneri*, 64.95% of which were serotype F2a. In the developed region, the only subtype of *Shigella* was *S*. *sonnei*. The proportion of five classes (EPEC, EAggEC, ETEC, EIEC and STEC) of DEC between the two regions showed no statistical significance (Fig 3).

Some of the cases were mixed infection that more than one pathogen had been detected positive from one sample simultaneously. For childhood cases in developed region, 38 cases were isolated bacteria and detected virus positive, 7 cases were isolated two or three bacteria and 45 cases were detected two or three virus positive. For childhood cases in developing region, 30 cases were isolated two or three bacteria. No cases had been detected 4 or more pathogens positive in both of two regions. The more details of pathogen list about mix infection were showed in Table 3.

**Table 3:** Pathogens of Mixed Infection in Diarrhea Cases.

	Children in Developed region	Adult in Developed region	Children in Developing region
**Bacteric+Virus**	**38**	**14**	**-**
DEC+ Rotavirus	4	2	
DEC+ Norovirus	9	5	
DEC+ Sapovirus	2	1	
DEC+Astrovirus		1	
DEC+ Adenovirus	9		
*Salmonella*+ Rotavirus	4	1	
*Salmonella*+Norovirus	3		
*Salmonella*+Astrovirus	2		
*Vibrio*+ Rotavirus		1	
DEC+*A*.*hydrophila*+Rotavirus	1		
DEC+Rotavirus+Norovirus	2		
DEC+Norovirus+ Sapovirus+	1		
DEC+ Norovirus+ Sapovirus		1	
DEC+ Norovirus+ Astrovirus		1	
*Salmonella*+Norovirus+ Sapovirus	1		
*Vibrio*+ Rotavirus+ Norovirus		1	
**Bacteria**	**7**	**10**	**30**
DEC+ *Salmonella*	5	1	2
DEC+ *Shigella*		2	7
DEC+ *Vibrio*		2	
DEC+*Y*.*enterocolitica*	1		1
DEC+*A*.*hydrophila*		2	
*Salmonella+Shigella*			4
*Salmonella*+*A*.*hydrophila*			6
*Shigella*+*Vibrio*		1	
*Shigella*+*A*.*hydrophila*			8
*Vibrio*+*A*.*hydrophila*		1	
*Vibrio*+*P*. *shigelloides*		1	
DEC+ *Salmonella*+*Shigella*			1
DEC+ *Salmonella*+*A*.*hydrophila*	1		1
**Virus**	**45**	**4**	**-**
Rotavirus+ Norovirus	7	1	
Rotavirus +Sapovirus	8	2	
Rotavirus+Astrovirus	3	1	
Rotavirus+ Adenovirus	6		
Norovirus+Sapovirus	5		
Norovirus +Astrovirus	3		
Norovirus+ Adenovirus	3		
Sapovirus+ Adenovirus	5		
Sapovirus +Astrovirus	2		
Rotavirus+Astrovirus+ Adenovirus	2		
Rotavirus+ Norovirus+ Sapovirus	1		

### Pathogen Spectrums between Children and Adults in Developed Regions

A total of 186 samples isolated one or more strains of 11 bacteria from 1047 adult cases. In 2014, 507 adult samples were also tested for five viruses, 120 of which were positive. Considering seasonal differences, the isolation rate was adjusted, showing that *Shigella*, EAggEC, EPEC, ETEC, *Vibrio*, *Yersinia*, rotavirus, sapovirus, astrovirus, and adenovirus were significantly different between the two populations of children and adults (Table 2). Multivariable regression analysis including covariates gender, onset season, and population showed that the isolation rate of adenovirus in children was 9.37 times higher than in adults (OR = 9.37; 95% CI:[3.41–25.72];*P* = 0.000), whereas *Vibrio* in adults was 33.86 times higher than in children (OR = 33.86; 95% CI: [4.56–251.12]; *P* = 0.001).

For the total spectrum contain virus and bacteria both (Fig 2B1 and 2B2), DEC (26.97%) and norovirus (23.65%) were the predominant adult diarrhea pathogens, followed by astrovirus, *Salmonella*, rotavirus, sapovirus, and *Vibrio*, which was distinctly different from children. For the virus spectrum (Fig 2C1 and 2C2), rotavirus (34.23%) were primary in children while norovirus (44.88%) for adults. Adenovirus showed the most discrepancy between two populations. For bacteria spectrum (Fig 2A1 and 2A3), DEC (56.63%) and *Salmonella* (17.86%) were primary in the adult bacterial spectrum, which was slightly lower than for children. However, the *Vibrio* rate in adults was more than 10 times higher than in children and *Shigella* was five times higher. Among 15 strains of *Shigella* isolated from adults, 12 were *S*. *sonnei* and three were *S*. *flexneri*. The distribution of five DEC in the two populations was sharply different. EPEC and EAggEC dominated infant and children diarrhea, and were seldom isolated from adults. ETEC was predominant in adults, whereas EPEC and EAggEC were rare (Fig 3).

About mixed infection of the adult cases in developed region, 14 cases were isolated bacteria and detected virus positive, 10 cases were isolated two bacteria and 4 cases were detected two or three virus positive (Table 3). There were no cases had been isolated 3 or more bacteria and none had been detected 4 or more virus positive neither.

### Pathogens and Clinical Symptoms of Infant and Childhood Diarrhea

Among the clinical symptoms in virus-positive cases, watery stool, fever, and vomiting were the primary characteristics and were especially typical in *Rotavirus*. Mucous or bloody stools and abnormal stools (WBC+ or RBC+) were routine features of bacterial diarrhea. *Salmonella* cases and *Shigella* cases shared similar symptoms. In addition, *Y*.*enterocolitica* and EPEC cases showed obviously abnormal stool routines (S3 Table).

## Discussion

The occurrence of bacterial diarrhea is closely related to the local economic development level [22–24]. Due to differences between social, economic and environmental factors characteristics, pathogen spectrums of infectious diarrhea vary. As the diversity of economic development level, people habits and customs, hygiene level between urban and countryside of China shows tremendous contrasts, which is distinct from European and American countries. The comparative study of infectious diarrhea pathogen spectrums between regions of different developmental levels was still relatively unknown in China before our study. However, we performed pathogen detection, including five viruses and 11 species of bacteria, on each sample from 3224 patients among three groups of people using the same method. This comparative study of the pathogen spectrum of infant infective diarrhea between a developed and developing region in China, between adults and infants in developed regions, is the first study of its kind. The primary difference in infant diarrhea pathogen spectrums between developed and developing regions in bacteriology was the detection rate of *Shigella* that was approximately 89 times, as the result of this study, higher compared to those in the developed region. The infant diarrhea spectrum of bacteria in developing regions in China was similar to those in Africa and South Asia [1, 7, 9, 10] and was correlated with the poverty and inadequate sanitation in Sui County in Henan. In the central urban region of Beijing, the levels of economic development and environmental conditions are similar to some developed countries which correlated with the spectrum of bacteria isolated, wherein *DEC*, *Salmonella*, and *Yersinia* were predominant and we rarely found *Shigella* [3, 5]. Furthermore, it was the first report that *Yersinia enterocolitica* infections were presence in Beijing.

We speculate that the different pathogen spectrums between children and adults in Beijing were caused by the more complex environment to which the adults were exposed. The activity space of infants and young children in the city is comparatively clean and limited, so the pathogen species frequency is relatively low (Fig 2). The adult activity range is larger and they are more likely to come into contact with many kinds of contaminated foods as potential sources of infections. Moreover, there are a large number of rural migrant workers from poorer provinces living in Beijing and they live with poorer health conditions and environments. These cases were a portion of the clinical cases so the pathogenic spectrums of adults are more complicated than those of infants and young children in the city.

In this study, isolated the suspicious strains first and then detected the virulence gene [20] from the DEC to identify and classify the strain. The data indicate three groups of people were found with a higher DEC detection rate. Continuous surveillance confirmed that DEC was the common cause of diarrhea in different groups of people in China. This project completes and corrects the deficiency of long-term DEC detection in China. This study identified species difference between the adults and children: ETEC was predominant in adults, whereas EPEC and EAggEC were the primary species in children. And no significant relationship between the local levels of development with DEC categories was found (Fig 3). Since the outbreak of EHEC O157: H7 that occurred in Xuzhou of Jiangsu Province, in 2000[25], it is generally considered that STEC caused severe symptoms in China. And STEC had not been reported in diarrhea patients in Beijing until our findings. Some of infants and young children infected with STEC in Beijing were verified firstly presented in this study, which with milder symptoms rather than severe symptoms and death.

**Fig 4:**
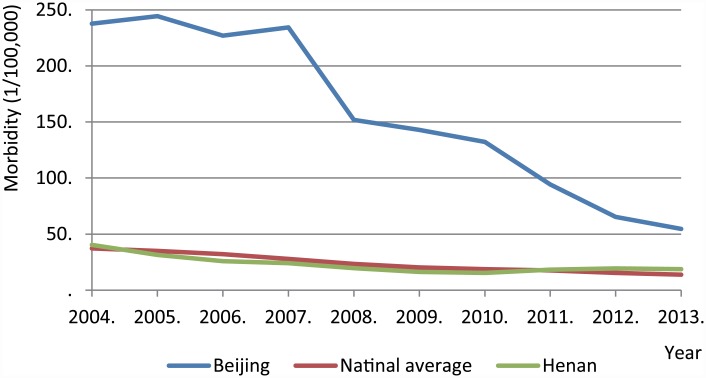
The morbidity of *Shigella* (symptoms diagnosed) reported by clinicians.

At present, only rotavirus and norovirus can be confirmed by etiological diagnosis in some large hospitals in China; many other diarrheal pathogens are diagnosed by clinical symptoms. *Shigella*, for example, is often diagnosed according to stool characteristics with pus, blood, or mucous test results such as being WBC and RBC positivity and symptoms like tenesmus. Through our correlative analysis of diarrhea pathogens and clinical symptoms, this study suggests that such a diagnosis method makes it hard to differentiate between *Shigella*, *Salmonella*, and *Yersinia* infections. From 2004 to 2013, the notifiable infectious disease reports of China, which acquired from the Data-Center of China Public Health Science (http://www.phsciencedata.cn/Share/en/index.jsp), suggested that morbidity from *Shigella* in Beijing was ranked in first place for ten years, except in 2013 (occupying second), which was 300%–600% higher than the national average (Fig 4). This differs from the data represented in this study, which showed that the detection rate for *Shigella* in infants and young children in the developing region was far higher than that in the developed region. We conclude that clinical diagnosis without etiologic diagnosis gives rise to the deviation in reports.

## Conclusion

We investigated childhood diarrhea in the developed region of China for three years consecutively and compared to diarrheal adults in the developed region and children in the developing region. It is the first time to assess the etiological diversity of childhood infectious diarrhea between two regions in China. The study showed a large difference in the etiology of bacterial diarrhea between children in developing and developed regions and the most obvious diversity was *Shigella*. There was no significant relationship between the local levels of development with DEC categories, but the age it was. EPEC and EAggEC were the primary classes of DEC in children from both regions, whereas it was ETEC in adults. *Shigella* and *Salmonella* induced similar symptoms in children, which may lead to the misidentification of notifiable diseases. *Y*.*enterocolitica* and STEC infections were discovered for the first time in Beijing.

## Supporting Information

S1 Table The sequences of the Specific Primers Used in RT-PCR to Detect Rotavirus, Adenovirus, Astrovirus, Norovirus, and Sapovirus. (DOCX)

S2 Table The sequences of the Specific Primers Used in PCR to classify EPEC, ETEC, EAggEC, EIEC and STEC. (DOCX)

S3 Table The association of clinical symptoms with pathogen detection positive. (DOCX)
