# Supplementary material for: Etiology of Childhood Infectious Diarrhea in a Developed Region of China: Compared to Childhood Diarrhea in a Developing Region and Adult Diarrhea in a Developed Region
Source: PLoS One. 2015 Nov 3;10(11):e0142136. doi: 10.1371/journal.pone.0142136 (PMC4631449; doi:10.1371/journal.pone.0142136)
Supplement: S1 Table — (DOCX) [file pone.0142136.s001.docx]

S1 Table. The sequences of the Specific Primers Used in RT-PCR to Detect Rotavirus, Adenovirus, Astrovirus, Norovirus, and Sapovirus

| Virus | Target | Primer name | Polarity | Sequence (5’–3’) | Amplicon size (bp) |
| --- | --- | --- | --- | --- | --- |
| Group A rotavirus | VP7 | Beg9 | + | GGCTTTAAAAGAGAGAATTTCCGTCTGG | 395 |
|  |  | VP7-1 | - | ACTGATCCTGTTGGCCATCCTTT |  |
| Group B rotavirus | VP7 | B5-2 | + | GGCAATAAAATGGCTTCATTGC | 814 |
|  |  | B5-3 | - | GGGTTTTTACAGCTTCGGCT |  |
| Group C rotavirus | VP7 | NG8S1 | + | ATTATGCTCAGACTATCGCCAC | 352 |
|  |  | NG8S2 | - | GTTTCTGTACTAGCTGGTGAAC |  |
| Adenovirus | Hexon | Ad1 | + | TTCCCCATGGCICAYAACAC | 482 |
|  |  | Ad2 | - | CCCTGGTAKCCRATRTTGTA |  |
| Astrovirus | Capsid | Precap1 | + | GGACTGCAAAGCAGCTTCGTG | 719 |
|  |  | 82b | - | GTGAGCCACCAGCCATCCCT |  |
| Norovirus GI | Capsid | G1-SKF | + | CTGCCCGAATTYGTAAATGA | 330 |
|  |  | G1-SKR | - | CCAACCCARCCATTRTACA |  |
| Norovirus GII | Capsid | COG2F | + | CARGARBCNATGTTYAGRTGGATGA | 387 |
|  |  | G2-SKR | - | CCRCCNGCATRHCCRTTRTACAT |  |
| Sapovirus | Capsid | SLV-5317 | + | CTCGCCACCTACRAWGCBTGGTT | 434 |
|  |  | SLV-5749 | - | CGGRCYTCAAAVSTACCBCCCCA |  |
| Norovirus | Polymerase | P290 | + | GATTACTCCAAGTGGGACTCCAC | 331 |
|  |  | P289 | - | TGACAATGTAATCATCACCATA |  |
