# Supplementary material for: Etiology of Childhood Infectious Diarrhea in a Developed Region of China: Compared to Childhood Diarrhea in a Developing Region and Adult Diarrhea in a Developed Region
Source: PLoS One. 2015 Nov 3;10(11):e0142136. doi: 10.1371/journal.pone.0142136 (PMC4631449; doi:10.1371/journal.pone.0142136)
Supplement: S2 Table — (DOCX) [file pone.0142136.s002.docx]

S2 Table. The sequences of the Specific Primers Used in PCR to classify EPEC, ETEC, EAggEC, EIEC and STEC*

| Gene & Primer name | Sequence (5'–3') | Amplicon size (bp) |
| --- | --- | --- |
| *stx1*-F | CAGTTAATGTGGTGGCGAAGG | 348 |
| *stx1*-R | CACCAGACAATGTAACCGCTG |  |
| *stx2*-F | ATCCTATTCCCGGGAGTTTACG | 584 |
| *stx2*-R | GCGTCATCGTATACACAGGAGC |  |
| *eae*-F | TCAATGCAGTTCCGTTATCAGTT | 482 |
| *eae*-R | GTAAAGTCCGTTACCCCAACCTG |  |
| *bfp*-F | GGAAGTCAAATTCATGGGGGTAT | 300 |
| *bfp*-R | GGAATCAGACGCAGACTGGTAGT |  |
| *lt*-F | GCACACGGAGCTCCTCAGTC | 218 |
| *lt*-R | TCCTTCATCCTTTCAATGGCTTT |  |
| *stII*-F | AAAGGAGAGCTTCGTCACATTTT | 129 |
| *stII*-R | AATGTCCGTCTTGCGTTAGGAC |  |
| *virF*-F | AGCTCAGGCAATGAAACTTTGAC | 618 |
| *virF*-R | TGGGCTTGATATTCCGATAAGTC |  |
| *ipaH*-F | CTCGGCACGTTTTAATAGTCTGG | 933 |
| *ipaH*-R | GTGGAGAGCTGAAGTTTCTCTGC |  |
| *aafII*-F | CACAGGCAACTGAAATAAGTCTGG | 378 |
| *aafII*-R | ATTCCCATGATGTCAAGCACTTC |  |

*EPEC: *eae* positive or *bfp* positive;

ETEC: *lt* positive or *stII* positive;

EAggEC: *aafII* positive;

EIEC: *virF* positive and *ipaH* positive;

STEC: *stx1* positive or *stx2* positive.
