# Supplementary material for: Etiology of Childhood Infectious Diarrhea in a Developed Region of China: Compared to Childhood Diarrhea in a Developing Region and Adult Diarrhea in a Developed Region
Source: PLoS One. 2015 Nov 3;10(11):e0142136. doi: 10.1371/journal.pone.0142136 (PMC4631449; doi:10.1371/journal.pone.0142136)
Supplement: S3 Table — (DOCX) [file pone.0142136.s003.docx]

S3 Table. The association of clinical symptoms with pathogen detection positive

| Pathogen | Rate of Symptoms(%) and *P* value* | | | | | | | | | | | | | | | |
| --- | --- | --- | --- | --- | --- | --- | --- | --- | --- | --- | --- | --- | --- | --- | --- | --- |
|  | Watery stool | | Mucus stool | | Bloody stool | | Loose stool | | Vomit | | Fever | | WBC | | RBC | |
| Virus | 73.77 | 0.000 | 1.28 | 0.155 | 0.21 | 0.051 | 24.73 | 0.000 | 22.39 | 0.000 | 11.94 | 0.010 | 17.99 | 0.000 | 3.00 | 0.000 |
| Rotavirus | 83.80 | 0.000 | 1.68 | 0.713 | 0.56 | 0.593 | 13.97 | 0.000 | 30.17 | 0.000 | 20.11 | 0.000 | 12.36 | 0.000 | 2.81 | 0.035 |
| Norovirus | 67.63 | 0.157 | 0.72 | 0.246 | 0.00 | 0.233 | 31.65 | 0.391 | 22.30 | 0.000 | 7.91 | 0.597 | 17.39 | 0.000 | 1.45 | 0.012 |
| Sapovirus | 70.31 | 0.166 | 0.00 | 0.238 | 0.00 | 0.432 | 29.69 | 0.366 | 21.88 | 0.004 | 4.69 | 0.206 | 17.19 | 0.003 | 3.13 | 0.271 |
| Astrovirus | 75.00 | 0.061 | 2.08 | 0.983 | 0.00 | 0.498 | 22.92 | 0.075 | 14.58 | 0.415 | 14.58 | 0.183 | 16.67 | 0.008 | 2.08 | 0.212 |
| Adenoviridae | 64.52 | 0.619 | 3.23 | 0.402 | 0.00 | 0.338 | 32.26 | 0.573 | 12.90 | 0.537 | 9.68 | 0.853 | 27.96 | 0.174 | 4.30 | 0.388 |
| Bacteria | 32.06 | 0.000 | 10.53 | 0.011 | 32.06 | 0.000 | 25.36 | 0.243 | 12.68 | 0.037 | 16.75 | 0.246 | 47.98 | 0.000 | 12.63 | 0.000 |
| *Salmonella* | 41.58 | 0.206 | 9.90 | 0.367 | 18.81 | 0.610 | 29.70 | 0.637 | 15.84 | 0.956 | 21.78 | 0.048 | 55.74 | 0.000 | 18.03 | 0.000 |
| *Shigella* | 3.82 | 0.000 | 16.79 | 0.000 | 74.81 | 0.000 | 4.58 | 0.000 | 8.40 | 0.014 | 21.37 | 0.033 | 100.00 | 0.017 | 66.67 | 0.000 |
| EAggEC | 43.06 | 0.420 | 4.17 | 0.266 | 20.83 | 0.372 | 31.94 | 0.408 | 12.50 | 0.405 | 16.67 | 0.674 | 34.00 | 0.948 | 8.00 | 0.643 |
| EPEC | 39.56 | 0.111 | 7.69 | 0.967 | 12.09 | 0.207 | 40.66 | 0.005 | 8.79 | 0.054 | 14.29 | 0.860 | 48.44 | 0.016 | 9.38 | 0.324 |
| ETEC | 57.14 | 0.617 | 0.00 | 0.448 | 14.29 | 0.851 | 28.57 | 0.957 | 0.00 | 0.247 | 0.00 | 0.267 | 40.00 | 0.793 | 20.00 | 0.215 |
| EIEC | 50.00 | 0.911 | 0.00 | 0.482 | 16.67 | 0.985 | 33.33 | 0.755 | 16.67 | 0.967 | 16.67 | 0.905 | 20.00 | 0.496 | 0.00 | 0.557 |
| EHEC | 57.14 | 0.617 | 0.00 | 0.448 | 0.00 | 0.231 | 42.86 | 0.368 | 0.00 | 0.247 | 0.00 | 0.267 | 40.00 | 0.793 | 0.00 | 0.557 |
| *Yersinia* | 33.33 | 0.386 | 11.11 | 0.688 | 0.00 | 0.174 | 55.56 | 0.061 | 0.00 | 0.189 | 11.11 | 0.747 | 100.00 | 0.000 | 4.00 | 0.000 |
| *A. hydrophila* | 38.24 | 0.264 | 20.59 | 0.004 | 29.41 | 0.051 | 11.76 | 0.037 | 23.53 | 0.230 | 8.82 | 0.314 | 28.57 | 0.743 | 0.00 | 0.487 |

*: The figures in shadow show a significant *P* value in the Chi2 test comparing the symptoms rate which the case pathogen detected positive greater than the rate which the case pathogen detected negative.
